# Supplementary material for: Monitoring Genetic Erosion of Aromatic and Medicinal Plant Species in Alentejo (South Portugal)
Source: Plants (Basel). 2023 Jul 8;12(14):2588. doi: 10.3390/plants12142588 (PMC10386371; doi:10.3390/plants12142588)
Supplement: Supplementary file 1 [file plants-12-02588-s001.zip › plants-2477192-supplementary.pdf]

plants

Table S1. Survey formulary used during 2011 resampling missions for each cultivated accession.

Plant species	CS, MC or MP
Accession code	*
GPS coordinates	
1.1 – Finding the person (farmer)	
Yes	
No	
1.2 - If not:	
Died	
Migrated	
2.1 – Continue to use the plant?	
Yes	
No	
2.2 If not:	
for how many years ago (n.º)	
lost the seed	
abandoned the activity	
buy the seed	
buy the plant	
causes (text)	
gave seed to neighbours (yes)	
Field notes	

CS - *Coriandrum sativum*, MC- *Mentha cervina*, MP - *Mentha pulegium*, * 2002/2003 field missions: 28 accessions of MC; 19 accessions of MP; 30 accessions of CS plus 18 accessions (from a total of 34) collected previously from BPGV – Portuguese Plant Germplasm Banc.

Table S2. Survey formulary used during 2011 resampling missions for each wild species accession.

Plant species	MC or MP
Accession code	*
GPS coordinates	
3.1 - Accession remains in the same location	
Yes	
No	
3.2 - relative abundance	
maintenance	
reduction	
increase	
3.3 - Genetic erosion causes	
river cleaning	
habitat destruction	
invasive by weeds	
mechanization and herbicides	
grazing	
overharvest	
desertification	
Field notes	

MC- *Mentha cervina*, MP - *Mentha pulegium*, * MC with 6 wild accessions; MP with 22 locations.
Note: some MP locations had cultivated and also wild plants nearby.
